# Beta-Blocker Use in Patients Undergoing Non-Cardiac Surgery: A Systematic Review and Meta-Analysis

**DOI:** 10.3390/medsci12040064

**Published:** 2024-11-11

**Authors:** Doménica Herrera Hernández, Bárbara Abreu, Tania Siu Xiao, Andreina Rojas, Kevin López Romero, Valentina Contreras, Sol Villa Nogueyra, Zulma Sosa, Samantha M. Alvarez, Camila Sánchez Cruz, Ernesto Calderón Martinez

**Affiliations:** 1 Facultad de Medicina, Pontificia Universidad Católica del Ecuador, Quito 170525, Ecuador; 2 Facultad de Medicina, Universidad de Carabobo, Valencia 2005, Venezuela; 3 Radiology Department, Thomas Jefferson University, Philadelphia, PA 19107, USA; 4 Facultad de Medicina, Universidad de Oriente, Núcleo Anzoátegui, Barcelona 6001, Venezuela; 5 Facultad de Medicina, Universidad del Salvador, San Salvador 1101, El Salvador; 6 Department of Lymphoma and Myeloma, MD Anderson Cancer Center, Houston, TX 77030, USA; 7 Facultad de Medicina, Universidad de Buenos Aires, Buenos Aires C1053ABK, Argentina; 8 Facultad de medicina, Universidad Evangélica de El Salvador, San Salvador 1101, El Salvador; 9 Faculty of Medicine, Saint George’s University, True Blue FZ818, Grenada; 10 Facultad de Medicina, Universidad Nacional Autónoma de México, Ciudad de México 04510, Mexico; mc21sacc2298@facmed.unam.mx; 11 Department of Internal Medicine, The University of Texas Health Science Medical Center, Houston, TX 77030, USA

**Keywords:** perioperative beta-blockers, non-cardiac surgery, myocardial infarction, mortality, hypotension, bradycardia, stroke, cardiovascular outcomes

## Abstract

Background: The use of beta-blockers in the perioperative period has been widely investigated due to their potential to reduce the risk of major adverse cardiovascular and cerebrovascular events (MACCE) and mortality; yet their overall impact on various postoperative outcomes remains debated. This study constitutes a systematic review and meta-analysis of the impact of beta-blockers on mortality, MI, stroke, and other adverse effects such as hypotension and bradycardia in patients undergoing non-cardiac surgery. Methods: A comprehensive systematic review and meta-analysis were conducted according to PRISMA 2020 guidelines. Searches were performed across PubMed, Cochrane, Web of Science, Scopus, EMBASE, and CINAHL databases; we included randomized controlled trials and cohort and case-control studies published from 1999 to 2024. Results: This meta-analysis included data from 28 studies encompassing 1,342,430 patients. Perioperative beta-blockers were associated with a significant increase in stroke risk (RR 1.42, 95% CI: 1.03 to 1.97, *p* = 0.03, I^2^ = 62%). However, no statistically significant association was found between beta-blocker use and mortality (RR 0.62, 95% CI: 0.38 to 1.01, *p* = 0.05, I^2^ = 100%). Subgroup analyses revealed a protective effect on mortality for patients with high risks, such as patients with a history of atrial fibrillation, chronic heart failure, and other arrhythmias. For myocardial infarction (RR 0.82, 95% CI: 0.53 to 1.28, *p* = 0.36, I^2^ = 86%), a reduction in events was observed in the subgroup of randomized controlled trials. Beta-blockers significantly increased the risk of hypotension (RR 1.46, 95% CI: 1.26 to 1.70, *p* < 0.01, I^2^ = 25%) and bradycardia (RR 2.26, 95% CI: 1.37 to 3.74, *p* < 0.01, I^2^ = 64%). Conclusions: Perioperative beta-blockers show increasing rates of stroke events following non-cardiac surgery but do not significantly impact the incidence of MI or mortality. The increased risks of hypotension and bradycardia necessitate careful patient selection and monitoring. Future research should aim to refine patient selection criteria and optimize perioperative management to balance the benefits and risks of beta-blocker use in surgical settings.

## 1. Introduction

Approximately 17.2 million surgical procedures are performed annually in the United States [1,2], and global surgical volume has surged dramatically over the past few decades, escalating from an estimated 300 million in 2012 [3,4]. It is crucial to highlight that nearly every surgical intervention carries inherent risks, and non-cardiac surgeries are no exception. Non-cardiac surgical interventions have been correlated with major adverse cardiovascular and cerebrovascular events (MACCE), occurring in up to 3% of the patients undergoing non-cardiac procedures [5].

Perioperative major adverse cardiac events (MACE) are defined as those occurring within 30 days after surgery. MACEs encompass conditions such as ischemic heart disease, stroke, transient ischemic attack, arrhythmias, heart failure, cardiac arrest, and cardiovascular death. Myocardial infarction (MI) and ischemic stroke in particular constitute a significant source of perioperative morbidity and mortality. [6,7].

The incidence of cardiovascular complications following non-cardiac surgical procedures appears to be multifactorial. The physiological changes during the perioperative period place significant stress on the cardiovascular system, with elevated levels of inflammatory cytokines such as tumor necrosis factor-alpha, interleukin (IL)-1, IL-6, and C-reactive protein (CRP) [4,6]. These changes trigger increased cardiac stress, altered hemodynamics, which, hence, affect coronary artery perfusion, ventricular and/or valvular function, and, finally, compromise of the cardiac conduction system, as normal homeostatic responses begin to fail [8].

Therefore, perioperative assessment and management of the cardiovascular system are emphasized in several society guidelines, including the 2014 guidelines from the American College of Cardiology and American Heart Association (ACC/AHA), the European Society of Anesthesiology and European Society of Cardiology (ESA/EHA), and the 2017 guidelines from the Canadian Cardiovascular Society (CCS) [9].

Given the critical impact of perioperative cardiovascular complications, the use of beta-blockers in the perioperative period has been investigated as a strategy to reduce the incidence of MACCE in patients undergoing non-cardiac surgery. The most recent guidelines, as stipulated in the ACC/AHA guidelines of 2014, propose that beta-blocker usage may be deemed reasonable under specific clinical scenarios to prevent the mentioned events. However, few data support its effectiveness and risk of surgical death reduction [10].

Beta-blockers are known for their ability to reduce heart rate, myocardial contractility, and blood pressure, potentially mitigating the cardiac stress induced by surgery [10,11,12]. Strong evidence indicates that beta-blockers may reduce the incidence of MI in patients undergoing non-cardiac surgery [13]. While this evidence suggests a beneficial effect of reducing MI, there is no significant difference in the occurrence of cerebrovascular events or ventricular arrhythmias.

However, these benefits come with potential risks, as beta-blockers may increase the incidence of bradycardia and hypotension. This highlights the need for careful consideration and monitoring when using beta-blockers in the perioperative setting to balance the potential cardiovascular benefits against the risks of adverse effects [8,13]. The data from the previous systematic reviews were derived from small trials with lower power, shedding light to fulfill the necessity of providing updated data extracted from the widely performed clinical trials.

Therefore, our study aimed to contribute to this understanding by systematically examining the impact of beta-blockers on mortality, MACCE, and stroke outcomes in non-cardiac surgery, analyzing data from recent large clinical trials and providing valuable insights to inform clinical practice and optimize perioperative management strategies.

## 2. Materials and Methods

The present study employed the Preferred Reporting Items for Systematic Review and Meta-analysis (PRISMA) 2020 guidelines to conduct a comprehensive systematic review [14,15]. The study was pre-registered on PROSPERO under ID: CRD42024548237.

### 2.1. Criteria for Considering Studies in This Review

#### 2.1.1. Types of Studies

We conducted a systematic review of randomized controlled trials (RCTs) and cohort and case-control studies written in English and Spanish, published from the inception of the database to October 2024 We excluded case reports, case series, protocol articles, reviews, and news articles. We also excluded duplicates and articles where relevant data extraction was not possible.

#### 2.1.2. Types of Participants

This study established specific participant selection criteria. It included adults of all genders, aged 18 and above, who underwent non-cardiac surgery and who may have chronic cardiac conditions such as atrial fibrillation and other arrhythmias. Exclusion criteria involved individuals <18 years old and patients who underwent cardiac surgeries.

#### 2.1.3. Type of Intervention

This study set specific intervention criteria for the use of beta-blockers with either placebo or standard treatments as comparators. Exclusion criteria involved interventions other than beta-blockers.

#### 2.1.4. Outcomes

Studies that report relevant outcomes, specifically related to short-term and long-term outcomes such as mortality, stroke (ischemic and hemorrhagic), MI (fatal and nonfatal), and hospitalization length of stay, were included in the study. Additionally, levels of hypotension (defined as systolic blood pressure <90 mmHg) and bradycardia (heart rate <60 bpm) were considered when reported as part of the outcomes in the articles. Studies reporting outcomes unrelated to cardiovascular complications were excluded.

#### 2.1.5. Search Method

We searched PubMed, Cochrane, Web of Science, Scopus, EMBASE, and CINAHL on 14 May 2024 using the following terms: beta-blockers, postoperative complications, and cardiovascular risk; for the specific searches per database, refer to our Supplementary Tables S1–S7. This study used a PRISMA flowchart to guide the selection of articles for the systematic review [14], resulting in a uniform dataset and enhancing the accuracy and reliability of our findings.

#### 2.1.6. Selection of Studies

Following an initial screening based on the title and abstract, two reviewers independently selected studies for inclusion using duplicates that were filtered first using Rayyan [16]. Consensus and consultation with a third reviewer were performed to resolve any reviewer discrepancies. Subsequently, a full-text analysis was conducted using the same methodology.

#### 2.1.7. Assessment of Risk of Bias

We evaluated the data using the criteria outlined in the Cochrane Handbook [17]. We applied the Risk of Bias 2.0 by Cochrane to assess the risk of bias in each randomized controlled trial [18] and New Castle Ottawa for cohort and case-control studies [19]. Two independent reviewers evaluated the risk of bias in each study, considering the specific criteria and guidelines provided by the respective tools. Any reviewer discrepancies were resolved through discussion with a third, blinded reviewer. Details regarding any downgrading or upgrading of evidence quality will be presented in the summary of findings table, providing transparency and explanations for bias assessment in each study included.

#### 2.1.8. Statistical Analysis

A meta-analysis was performed using R version 3.4.3 (R Core Team) [19,20]. The pooled effect of the outcomes was examined using a random effects meta-analysis (DerSimonian–Laird approach) [21]. Whenever the number of studies reporting an outcome of interest was insufficient, only a qualitative analysis of the results was performed [17]. Effect sizes were expressed as relative risk (RR) and 95% confidence interval. The I^2^ statistic assessed heterogeneity, and the following cut-off values were used for interpretation: <25, 25–50, and >50% were considered small, medium, and large heterogeneity, respectively [22]. For outcomes with medium and large heterogeneity, subgroup analysis and sensitivity analyses were performed according to the leave-one-out method to determine the influence of individual studies on the overall effect, using diagnostic plots proposed by Viechtbauer and Cheung [23]. The subgroup analysis examined the following variables: year, type of study design, country, type of surgery, risk of bias, type of medication, history of cardiovascular disease (CVD), history of chronic heart failure (CHF), history of atrial fibrillation, and history of other arrhythmias. A positive history of CVD was defined as either a direct report of CVD or the presence of at least three cardiac risk factors, including hypertension, high cholesterol, diabetes mellitus, parental history of premature MI, obesity, or current cigarette use. For the remaining variables, participants were classified as having the condition if this was present at baseline among some or all of the participants. Egger’s regression test examined publication bias when 10 or more reports with the same outcome were available [24]. Whenever possible, subgroup analyses were performed for primary outcomes.

## 3. Results

### 3.1. Systematic Review Results

**Figure 1:**
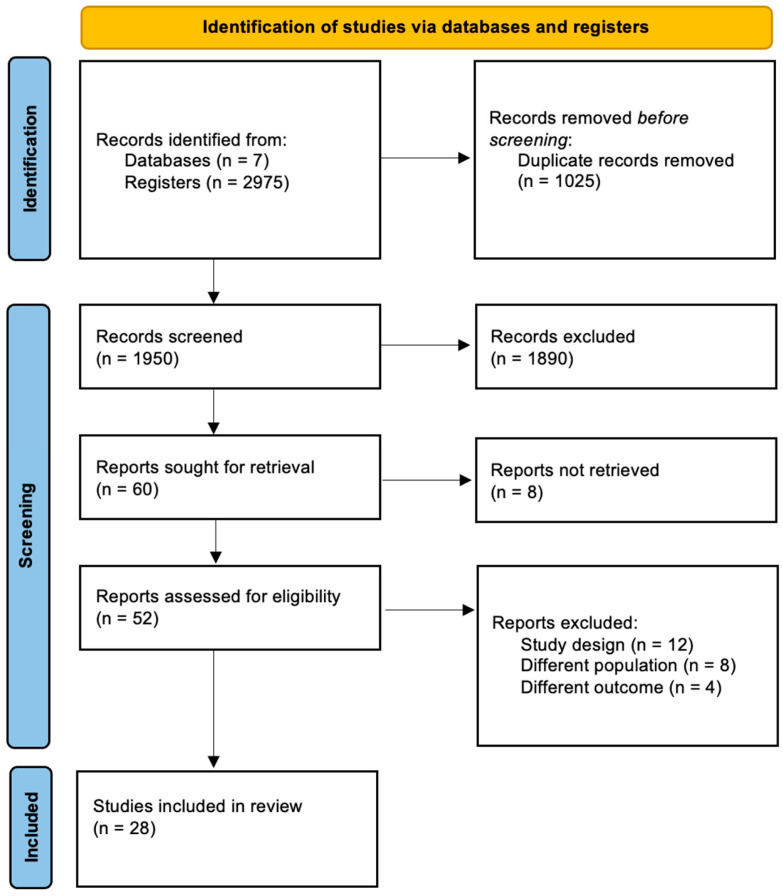
PRISMA flow chart. This flow diagram outlines the systematic process of identifying, screening, and including studies in this meta-analysis. A total of 2975 records were initially identified from seven databases, and 1024 duplicates were removed before screening. Out of the remaining 1950 records, 1890 were excluded during the screening process. Sixty reports were sought for retrieval, but eight were not retrieved, leaving 52 studies assessed for eligibility. Twenty-four studies were excluded due to study design (n = 12), wrong outcome (n = 8), or wrong population (n = 4), resulting in the inclusion of 28 studies in the final analysis.

A total of 2196 articles were collected from seven database searches. Before the screening of the potential articles, 247 duplicates were removed, and a total of 1949 records were sought for retrieval; 1890 were further removed in the screening process. Out of the remaining 60 publications, eight reports could not be retrieved. The resulting 52 reports were assessed for eligibility. A total of 24 of the 52 reports were excluded due to study design [12], different outcome [8], and different population [4]. A total of 28 articles were included. Figure 1 of the PRISMA flow diagram depicts the systematic approach adopted for study selection.

#### Study Characteristics

The primary outcome obtained from the selected research articles focused on assessing the effectiveness of the perioperative use of beta-blockers in reducing mortality, stroke, and MI in patients undergoing non-cardiac surgery, compared to patients who did not receive perioperative beta-blockers. Assessed articles included patients from around the world, including the USA (25%), Sweden (21.4%), the UK (17.8%), Canada (10.7%), Denmark (7.1%), The Netherlands (7.1%), Serbia (3.6%), Spain (3.6%), and Japan (3.6%). The total sample size included was 1,342,430 patients, with ages ranging from 32 to 74 years of age. A considerable number of studies (e.g., Martin et al., 2015 [25]; Lindenauer et al., 2005 [26]) had very large cohorts, which strengthened the generalizability of their, and our, findings. Study designs included were randomized control trials (RCTs), cohort studies, and case-control studies. Regarding the main findings, several studies reported that beta-blockers were associated with reduced mortality and cardiac events. From the studies included, 14 (50%) reported that beta-blockers reduced mortality rates, 7.1% reported an increase in mortality rates, 25% showed neutral outcomes in mortality, and 17.8% did not mention this outcome. While beta-blockers reduced MI in 30% of the studies, they also increased the risk of adverse effects like hypotension, bradycardia, and stroke in 40% of these studies. This information is summarized in the general outcomes in Table 1.

### 3.2. Risk of Bias

**Figure 2:**
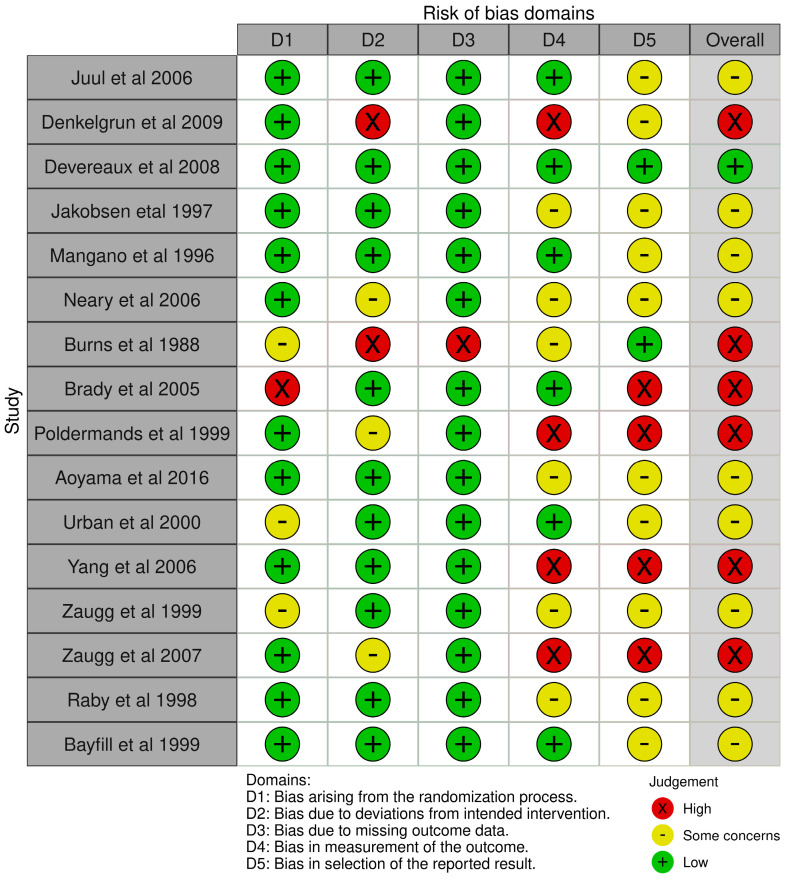
Risk of bias. This figure presents the risk of bias assessment for each study included in the analysis, covering five key domains: D1, bias arising from the randomization process; D2, bias due to deviations from intended intervention; D3, bias due to missing outcome data; D4, bias in the measurement of the outcome; and D5, bias in the selection of the reported result. Studies are color-coded to indicate the level of bias in each domain, with green representing low risk, yellow representing some concerns, and red indicating high risk. The overall judgment for each study was based on the cumulative assessment across all domains [32,33,34,35,37,38,41,42,43,45,46,48,49,50,51,52].

The risk of bias for the 28 included studies was assessed using the Newcastle–Ottawa Quality Assessment Scale (NOS) and the Cochrane Risk of Bias Tool (RoB 2.0). Twelve studies were evaluated with the NOS, revealing that all articles (100%) were of good quality, with a variation in total score from 7–9 points. This information is summarized in Table 2. Of the 16 studies assessed with RoB 2.0, one article (6.3%) had a low risk of bias, nine articles (56.2%) had some concerns, and six articles (37.5%) had a high risk of bias. This information is summarized in Figure 2.

### 3.3. Meta-Analysis Results

#### 3.3.1. Stroke

**Figure 3:**
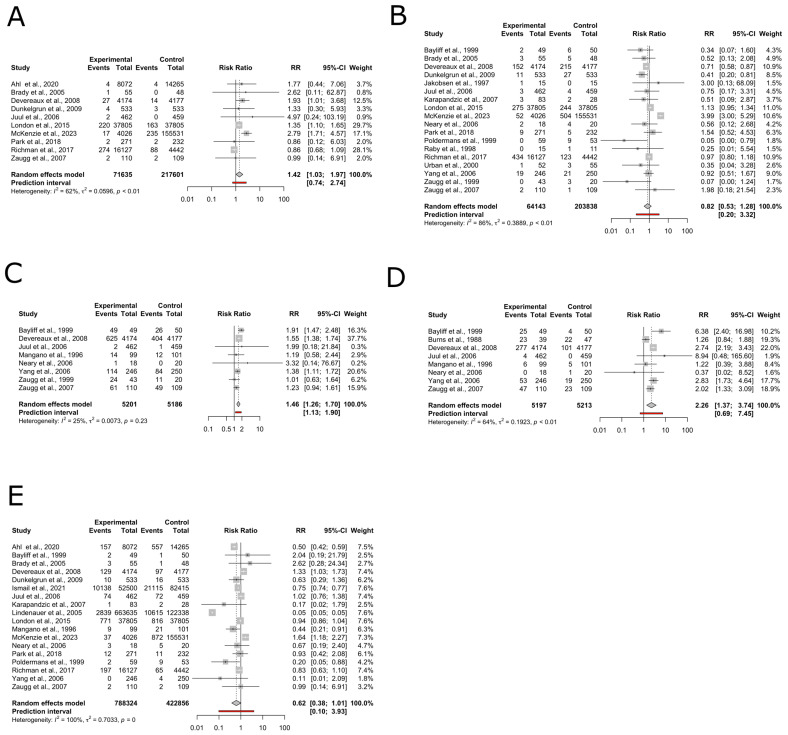
Forest plots. (**A**) Forest plot detailing relative risk and 95% confidence interval for the risk of stroke in the beta-blocker group compared to the non-beta-blocker group [25,27,28,32,33,34,38,40,42,44,51]. (**B**) Forest plot detailing relative risk and 95% confidence interval for the risk of MI in the beta-blocker group compared to the non-beta-blocker group [9,33,34,35,36,38,42,46,48,50,51]. (**C**) Forest plot detailing relative risk and 95% confidence interval for the risk of hypotension in the beta-blocker group compared to the non-beta-blocker group [19,32,34,37,38,49,50,51,52]. (**D**) Forest plot detailing relative risk and 95% confidence interval for the risk of bradycardia in the beta-blocker group compared to the non-beta-blocker group [9,32,34,37,38,41,42,49,50,51]. (**E**) Forest plot detailing relative risk and 95% confidence interval for the risk of mortality in the beta-blocker group compared to the non-betablocker group [19,26,27,32,33,34,36,38,40,41,42].

This meta-analysis evaluated the association between perioperative beta-blocker administration and stroke incidence in a total of 289,236 patients undergoing non-cardiovascular surgery. Data were obtained from 10 studies. The random effects model analysis revealed statistically significant association RR1.42 (95% CI: 1.03 to 1.97, *p* = 0.03, I^2^ = 62%). The prediction interval ranged from 0.74 to 2.74 (Figure 3A).

##### Subgroup and Sensitivity Analysis

Subgroup analyses showed significant differences in several areas. The analysis per year and type of surgery revealed differences (*p* < 0.01). However, they were based on groups with single studies, which limited reliability. Medication (*p* = 0.03), history of (CHF) (*p* < 0.01), and chronic use of beta-blocker (*p* < 0.01) also revealed significant differences. Metoprolol had a significant effect of RR, 2.03 (95% CI: 1.12 to 3.68, I2 = 0%). The subgroup of studies that did not report history of CHF had an RR of 2.79 (2.47 to 3.15, I^2^ = 0%). And studies that did not report chronic use of beta-blockers had an RR of 2.20 (1.52 to 3.19, I^2^ = 0%). Other subgroup analyses, including those based on country, type of study, history of atrial fibrillation, history of arrythmia, and risk of bias, did not show significant differences (Supplementary Table S8). A leave-one-out influence analysis identified two studies McKenzie et al., 2022 [28], and Richman et al., 2017 [44] as potential sources of heterogeneity (Supplementary Table S14). Upon analysis excluding these articles, we obtained an RR of 1.29 (95% CI: 1.22 to 1.59, *p* < 0.01, I^2^ = 0%) (Supplementary Table S9).

##### Publication Bias

**Figure 4:**
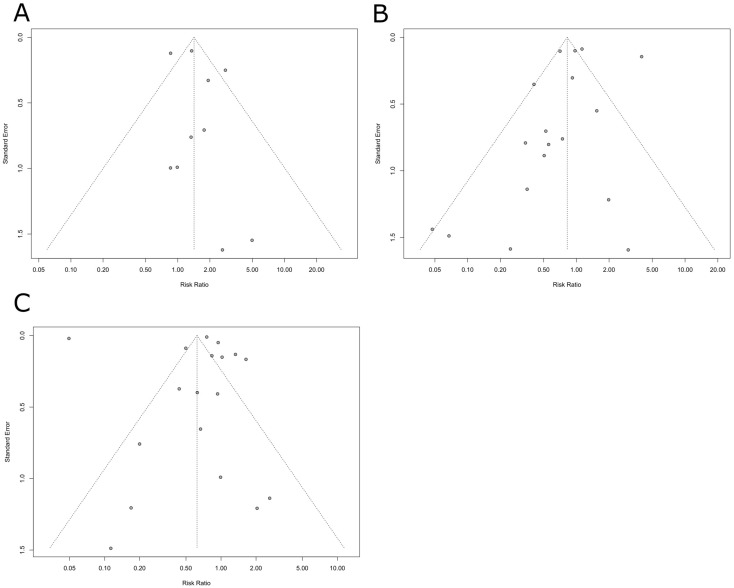
Funnel plots. (**A**) Funnel plot detailing publication bias in the included studies in a meta-analysis of the risk of stroke. (**B**) Funnel plot detailing publication bias in the included studies in a meta-analysis of the risk of MI. (**C**) Funnel plot detailing publication bias in the included studies in a meta-analysis of the risk of mortality.

The Egger test assessed funnel plot asymmetry, resulting in a t-value of a t = 0.97 (df = 8, *p* = 0.35), indicating no significant publication bias (Figure 4A).

#### 3.3.2. Myocardial Infarction

This meta-analysis evaluated the association between the use of perioperative beta-blockers and MI in a total of 267,981 patients. Data were obtained from 18 studies. The random effects model analysis demonstrated no statistically significant association, RR 0.82 (95% CI: 0.53 to 1.28, *p*-value = 0.36, I^2^ = 86%). The prediction interval ranged from 0.20 to 3.32 (Figure 3B).

##### Subgroup Analysis

Subgroup analyses revealed significant differences in the analysis by year (*p* < 0.01). However, most of the subgroups were based on single studies, which limited the reliability of these findings. Regarding the type of study, significant differences were found (*p* = 0.02), with RCTs showing a protective effect, RR 0.66 (95% CI: 0.51 to 0.84, I^2^ = 3.6%), and cohort studies showing a non-significant increased risk, RR 1.45 (95% CI: 0.60 to 3.54, I^2^ = 94.4%). In contrast, subgroup analyses based on country (*p* = 0.56), type of surgery (*p* = 0.58), risk of bias (*p* = 0.24), medication (*p* = 0.18), history of CVD (*p* = 0.97), history of CHF (*p* = 0.47), and history of atrial fibrillation or arrhythmia (*p* = 0.57 for both) did not show significant differences. Although these analyses did not reveal major variations, individual subgroups, such as those on metoprolol use, had a significant finding of RR 0.72 (95% CI: 0.62 to 0.84, I^2^ = 0%) (Supplementary Table S10). A leave-one-out influence analysis did not identify any specific study affecting the overall result (Supplementary Table S11).

##### Publication Bias

The funnel plot asymmetry suggested possible publication bias (Figure 4B). The Egger test assessed funnel plot asymmetry, though, resulting in a t-value of −0.77 (df = 16, *p* = 0.378), indicating no significant publication bias.

#### 3.3.3. Hypotension

This meta-analysis evaluated the association between the use of perioperative beta-blockers and hypotension in a total of 10,387 patients. Data were obtained from eight studies. The random effects model analysis demonstrated a statistically significant association, RR 1.46 (95% CI: 1.26 to 1.70, *p* < 0.01, I^2^ = 25%). The prediction interval ranged from 1.13 to 1.90 (Figure 3C).

##### Subgroup Analysis

Subgroup analysis was not possible due to the low heterogeneity among the studies.

##### Publication Bias

The funnel plot and the Egger test were not possible due to the low number of studies that analyzed this variable.

#### 3.3.4. Bradycardia

This meta-analysis evaluated the association between the use of perioperative beta-blockers and bradycardia in a total of 10,410 patients. Data were obtained from eight studies. The random effects model analysis demonstrated a statistically significant association (RR = 2.26, 95% CI: 1.37 to 3.74, *p* <0.01, I^2^ = 64%). The prediction interval ranged from 0.69 to 7.45 (Figure 3D)

##### Subgroup and Sensitivity Analysis

Subgroup analyses showed significant differences in the analysis by year, country, type of surgery, and medication (*p* < 0.01). However, most of the subgroups were based on single studies, which limited the reliability of these findings. Other subgroup analyses, including the history of CVD (*p* < 0.01), showed a significantly increased risk in those with a history of CVD (RR 2.66, 95% CI: 1.68 to 4.20). Subgroups such as risk of bias (*p* = 0.30), history of atrial fibrillation (*p* = 0.84), history of arrhythmia (*p* = 0.84), history of CHF (*p* = 0.92), and beta-blocker use (*p* = 0.89) did not reveal significant between-group differences, though certain individual subgroups like those with CVD and beta-blocker use demonstrated notable within-group effects (Supplementary Table S12). A leave-one-out influence analysis did not identify any specific study affecting the overall result (Supplementary Table S13).

##### Publication Bias

The funnel plot and the Egger test were not possible due to the low number of studies that analyzed this variable.

#### 3.3.5. Mortality

This meta-analysis evaluated the association between the use of perioperative beta-blockers and mortality in a total of 1,211,180 patients. Data were obtained from 18 studies. The random effects model analysis demonstrated a non-statistically significant association RR of 0.62 (95% CI: 0.38 to 1.01, *p* = 0.05, I^2^ = 100%). The prediction interval ranged from 0.10 to 3.93 (Figure 3E).

##### Subgroup and Sensitivity Analysis

Subgroup analyses revealed significant differences in the analysis by year and country (*p* < 0.01). However, most of the subgroups were based on single studies, which limited the reliability of these findings. A significant difference was seen in history of atrial fibrillation (AF) (*p* = 0.01), with those having AF showing a stronger protective effect (RR 0.32, 95% CI: 0.11 to 0.91, I^2^ = 99.4%). History of arrhythmia (*p* = 0.03) had a non-significant lower risk observed in patients with a history of arrhythmia (RR 0.29, 95% CI: 0.08 to 1.05, I^2^ = 98.9%). Finally, a history of CHF was a significant predictor of mortality benefit with beta-blocker (*p* = 0.05); those with CHF showed a protective effect (RR 0.54, 95% CI: 0.30 to 0.96, I^2^ = 99.9%) from beta-blocker. There were no significant differences by study type (*p* = 0.37), surgery type (*p* = 0.80), risk of bias (*p* = 0.80), or medication (*p* = 0.56) (Supplementary Table S14). A sensitivity analysis was pointed out by Lindenauer et al., 2005 [36], as a potential source of heterogeneity through leave-one-out analysis (Supplemental Table S15). However, after excluding this article, the analysis did not modify the previous results but considerably reduced the heterogeneity, obtaining an RR of 0.82 (95% CI: 0.63 to 1.08, *p* = 0.14, I^2^ = 83%).

##### Publication Bias

The funnel plot asymmetry suggests possible publication bias (Figure 4C). The Egger test assessed funnel plot asymmetry, resulting in a t-value of −0.10 (df = 16, *p* = 0.92), indicating no significant publication bias.

## 4. Discussion

This meta-analysis aimed to assess the efficacy and safety of perioperative beta-blocker use in non-cardiac surgery, focusing on the reduction in mortality, stroke, MI, and the presence of adverse effects like hypotension and bradycardia. During any type of surgery, the body experiences physiologic stress and high levels of catecholamines that are independently associated with an increased risk of death. Beta-blockers have been shown to decrease this physiological stress caused by the insult of major non-cardiac surgery by reducing the adrenergic receptor activation [27,53]. While their long-term benefit may stem from a reduction in postoperative mortality and cardiovascular adverse effects, it is crucial to acknowledge the associated risks, which must be carefully considered before initiating therapy [29,53,54,55].

Our analysis showed an increase stroke risk in the studied population, with overall findings achieving statistical significance, RR 1.42 (95% CI: 1.03 to 1.97, *p*-value = 0.03, I^2^ = 62%). In contrast, Devereaux et al., who included the POISE trial, showed that perioperative beta-blockers are associated with an increased risk of non-fatal stroke [33]. We can emphasize the consistency of our findings regarding stroke risk, despite variations in study designs and patient populations. Devereaux et al. reported that more patients with strokes were seen in the metoprolol group compared to the placebo group, with a hazard ratio of 2.17 (95% CI: 1.26–3.74, *p* = 0.0053) [34]. This result is consistent with our study and implies that the use of perioperative beta-blockers increases the risk of stroke during non-cardiac surgery.

According to the latest ACC/AHA guidelines, a perioperative beta-blockade before non-cardiac surgery prevents nonfatal MI [10]. This contrasts with our finding regarding MI, which demonstrated a non-statistically significant association, RR0.82 (95% CI: 0.53 to 1.28, *p*-value = 0.36 I^2^ = 86%). However, after a subgroup analysis regarding type of study due to heterogeneity, the RCTs showed a protective effect in MI (RR 0.66, 95% CI: 0.51 to 0.84, I^2^ = 3.6%), whereas the cohort studies showed a non-significant increased risk (RR 1.45, 95% CI: 0.60 to 3.54, I^2^ = 94.4%). Similar mixed results were observed by Alegria et al. (2019), who highlighted inconsistent outcomes from RCTs regarding perioperative beta-blockers and cardiovascular events, highlighting their rigorous design and minimized biases and controlled confounding variables, allowing for randomization; this aligns with current knowledge [56]. In contrast, cohort studies might have uncontrolled confounders, leading to varying results.

The analysis concerning adverse effects like hypotension and bradycardia revealed notable increased risks associated with perioperative beta-blocker administration. Our analysis showed that hypotension was significantly prevalent in patients receiving beta-blockers, with an RR of 1.46 (95% CI: 1.26 to 1.70, *p* < 0.01, I^2^ = 25%), emphasizing a potential clinical concern. Furthermore, beta-blockers were also linked to an increased risk of bradycardia requiring treatment, with an RR of 2.26 (95% CI: 1.37 to 3.74, *p* < 0.01, I^2^ = 64%), particularly evident in specific subgroups such as those with a history of CVD. It is crucial to interpret these results with awareness due to the high heterogeneity in the bradycardia subgroup. Key sources of heterogeneity included publication year, country of study, and type of surgery. However, this was expected as most studies come from the USA and diverse regions in Europe. The subgroup analysis revealed that studies including patients with a history of CVD showed an increased risk of bradycardia, RR 2.66 (95% CI: 1.68 to 4.20). This aligns with the findings of a meta-analysis conducted by Blessberger et al. in 2019, which also reported a statistically significant increase in bradycardia, in perioperative patients receiving beta-blockers (RR 2.49, 95% CI: 1.74 to 3.56), and hypotension (RR 1.40, In 95 In % CI 1.29 to 1.51) [13]. These findings align with clinical practice, where the cautious use of beta-blockers is essential to mitigate the risk of bradycardia [57].

Our findings underscore the role of perioperative beta-blockers in mortality among surgical patients, contrasting with previous research that reported a significant decrease in mortality rates across various RCTs. Our qualitative analysis showed that 40% of the studies reported a reduction in mortality rates, and our meta-analysis revealed a non-significant protective effect of perioperative betablockers (RR 0.62, 95% CI: 0.38 to 1.01 *p* = 0.05). However, significant association was found in subgroups with a history of atrial fibrillation, showing a protective effect in this group, RR 0.32 (IC 95% CI: 0.11 to 0.91 *p* = 0.01) and a history of arrhythmia, RR 0.29 (95% CI: 0.08 to 1.05); finally, our study showed a protective effect in those with a history of chronic heart failure, RR 0.54 (95% CI: 0.30 to 0.96). This aligns with results reported by Ziff et al. in 2020, which highlighted the substantial reduction in mortality in heart failure patients and further underscored the need to weigh benefits and risks [54]. The observed reduction in mortality in these specific groups aligns with the physiological advantages of beta-blockers, including their ability to provide rate control in arrhythmias, reduce myocardial oxygen demand via anti-ischemic effects, and improve diastolic filling [53,58]. These mechanisms address the key cardiovascular risks these conditions present during surgery. However, these findings should be interpreted with caution due to the high heterogeneity in mortality analyses. A previous meta-analysis conducted by Windle et al. in 2021 found no significant difference in early or long-term mortality with perioperative beta-blockers [59], reinforcing the need for individualized therapy. Differences in study designs and patient populations further underline the importance of careful patient selection and tailored perioperative management when using beta-blockers.

Our analysis showed a non-statistically significant reduction in MI and mortality, likely due to mixed outcomes in the RCTs and cohort studies. This aligns with findings from Ziff et al. (2020), who also reported similar variations in mortality across study types [55]. Notably, many RCTs lacked stratification between patients receiving perioperative beta-blockers for the first time and those with chronic use. This underscores the need for future clinical trials to focus on diverse populations and include further stratification to better clarify beta-blocker effects in these groups.

The ESC guidelines suggest that starting beta-blockers preoperatively may be considered for high-risk patients to reduce myocardial infarction incidence; our data from our RCT subgroup suggest a reduction in myocardial infarction in patients receiving beta-blockers, supporting this statement and providing evidence for a high-grade recommendation. On the other hand, the ESC strongly recommends the continuation of beta-blockers for patients who are already receiving this medication; our data support this recommendation in the context of mortality, as perioperative beta-blockers were shown to have a protective effect in specific subgroups. These findings suggest that the continuation of beta-blockers in these populations may confer survival benefits, aligning with the recommendations [60]. Our data highlight the importance of individualized patient selection, supporting the growing consensus that beta-blocker use should be tailored to patient-specific risk profiles and surgical contexts rather than applied broadly. Beta-blockers may be beneficial for patients with a history of atrial fibrillation, other arrhythmias, or chronic heart failure. However, there is currently no evidence from randomized controlled trials or individual-level data to guide these recommendations for different risk classifications in this context. Therefore, perioperative beta-blocker use is recommended for patients at high cardiovascular risk undergoing non-cardiac surgery. Our study underscores the need to stratify patients based on cardiovascular risk and to focus on specific populations, such as those with atrial fibrillation, other arrhythmias, or heart failure, to ensure appropriate beta-blocker use during the perioperative period.

## 5. Limitations

There are certain limitations to our study. First, the studies included in our analysis were highly heterogeneous in terms of design, sample size, population characteristics, and beta-blocker regimens. This significant heterogeneity in some outcomes limits the reliability of pooled estimates and suggests variability across studies. Second, the potential for publication bias is another limitation of our study, as indicated by the funnel plot asymmetry in the analysis of some outcomes, such as stroke and MI. Finally, our study focused on short-term outcomes without a comprehensive long-term evaluation of beta-blocker therapy on postoperative complications. Longer follow-up studies are required to evaluate the long-term effects of beta-blockers on cardiovascular events and mortality, and studies focusing on intermediate risk populations are needed in order to evaluate the effectivity of the intervention in this population.

Despite its limitations, this meta-analysis has several strengths. It incorporates a substantial sample size, with data derived from diverse geographical regions, enhancing the generalizability of the findings. Importantly, we conducted several subgroup analyses, some of which had not been performed in previous meta-analyses. These new analyses provide new insights into factors that may influence outcomes, such as the type of beta-blocker, CHF history, and previous cardiac diseases. Furthermore, our sensitivity analyses helped identify studies contributing to heterogeneity and demonstrated that excluding these studies did not significantly alter the main conclusions. This strengthens the confidence in the overall findings of this updated meta-analysis.

## 6. Conclusions

This meta-analysis provides valuable insights into the perioperative use of beta-blockers in non-cardiac surgery, highlighting both their benefits and associated risks. While beta-blockers were found to significantly increase the risk of stroke, they did not demonstrate a statistically significant reduction in MI or mortality across the overall study population. However, subgroup analyses revealed protective effects in specific populations, such as those with atrial fibrillation and chronic heart failure. Notably, the risk of adverse events, such as hypotension and bradycardia, was also elevated, particularly in patients with a history of cardiovascular disease.

The findings emphasize the importance of carefully considering patient-specific cardiovascular risks before initiating perioperative beta-blocker therapy. The heterogeneity observed across studies, particularly between randomized control trials (RCTs) and cohort studies, suggests the need for future research that includes better stratification of patient populations and more rigorous study designs. Despite these limitations, the large sample size and geographical diversity of the included studies enhance the generalizability of the findings. Future studies should also aim to assess the long-term effects of beta-blockers on postoperative outcomes, as this meta-analysis primarily focused on short-term outcomes.

## Figures and Tables

**Table 1:** General outcomes.

Author(s)	Year	Country	Study Design	Sample Size (Total)	Cases	Control	Mean ± SD Age	Beta-Blocker Use Definition	
Ahl et al., 2020 [27]	2020	Sweden	Cohort study	22,337	8072	14,265	70	Patients within beta-blocker use 12 months preoperatively	Preoperative beta-blockers are strongly associated with reduced short-term and long-term mortality following colon cancer surgery.
McKenzie et al., 2022 [28]	2022	USA	Retrospective cohort study	204,981	45,424	155,531	55.1	Patients with preoperative use of beta- blocker within 60 days before surgery	No association between preoperative beta-blocker and chronic beta-blocker use was observed regarding stroke risk.
Ahl R. et al., 2019 [29]	2018	Sweden	Prospective cohort study	11,966	3513	8453	NA	Prescription of beta-blockers prior to surgery	Preoperative beta-blocker exposure is associated with a decrease in 30-day mortality. Improved survival was observed with the use of beta-blockers before surgery.
Ahl et al., 2019 [30]	2019	Sweden	Cohort study	3139	671	2468	73.1 ± 12.4	Prescription of beta-blockers prior to surgery	Beta-blocker use linked to a 60% risk reduction in 1-year mortality. No significant association with reductions in major complications.
Ismail et al., 2021 [31]	2021	Sweden	Retrospective cohort study	134,915	52,493	82,408	NA	Prescription of beta-blockers 12 months before and after the surgery	Significant reduction in the risk of mortality in the first year following hip fracture surgery with ongoing beta-blocker therapy.
Juul et al., 2006 [32]	2006	Denmark	Randomized controlled trial	921	462	459	NA	Prescription perioperatively and maximum 8 days	Metoprolol did not significantly affect mortality and cardiac morbidity in patients with diabetes.
Dunkelgrun et al., 2009 [33]	2009	Netherlands	Randomized controlled trial	1066	264	268	NA	Prescription prerioperative with a median of 34 days before surgery and continued 30 days after surgery	Bisoprolol was associated with a significant reduction in 30-day cardiac death and nonfatal MI.
Devereaux et al., 2008 [34]	2008	Canada	Randomized controlled trial	9298	4174	4177	NA	Prescription of betab-lockers 2–4 h before surgery and continued 30 days	Metoprolol use prevented perioperative MI. Increased 30-day risk of total mortality and stroke was observed.
Jakobsen et al., 1997 [35]	1997	Denmark	Prospective, Randomized	30	15	15	NA	Prescribed beta-blockers 1 day before and during observational period	Perioperative oral beta-blockade can reduce the frequency of atrial fibrillation without serious side effects.
London et al., 2015 [25]	2015	UK	Retrospective cohort study	136,745	55,138	81,607	64.4 ± 10.2	Prescribed beta-blockers on day 0 or 1 postoperatively	Perioperative beta-blocker exposure was associated with lower rates of 30-day all-cause mortality in patients with two or more Revised Cardiac Risk Index factors.
Karapandzic et al., 2007 [36]	2007	Serbia	Prospective cohort study	111	83	28	64 ± 33.3	Chronic use of beta-blockers maintained before and after surgery	Perioperative cardio protection significantly reduced mortality until postoperative day 30 in patients having open abdominal nonvascular surgery with general anesthesia.
Lindenauer et al., 2005 [26]	2005	USA	Retrospective cohort study	782,969	663,635	122,338	68	Prescribed beta-blockers on day 1 or 2 of hospitalization	Perioperative beta-blocker therapy is associated with a reduced risk of in-hospital death among high-risk patients undergoing major non-cardiac surgery. Patient safety may be enhanced by increasing the use of beta-blockers in high-risk patients.
Mangano et al., 1996 [37]	1996	USA	Randomized controlled trial	200	99	101	NA	Prescribed beta-blockers 1 day before surgery and maximum 8 days	Treatment with atenolol during hospitalization can reduce mortality and the incidence of cardiovascular complications in patients with risk of cardiovascular disease undergoing non-cardiac surgery. Efficacy and safety of perioperative beta-blockade demonstrated even for patients with a history of heart failure and pulmonary disease
Neary et al., 2006 [38]	2006	UK	Randomized controlled trial	38	18	20	NA	Prescribed beta-blockers 1 day before surgery and maximum 8 days	Due to the high rate of follow-up loss, no significant outcomes were shown.
Noorzidj et al., 2007 [39]	2007	The Netherlands	Case-control study	2868	989	1879	NA	Peioperative use of beta-blockers	Perioperative beta-blockade was associated with significantly reduced in-hospital mortality rates in higher-risk subgroups of the total study. In contrast, increased in-hospital mortality rates among the lowest-risk patients.
Park et al., 2018 [40]	2018	Spain	Retrospective cohort study	503	271	232	NA	Patients with prescribed bet a-blocker use before surgery	Preoperative beta-blocker therapy may not be associated with postoperative clinical outcomes in coronary revascularized patients without severe LVSD or CHF.
Burns et al., 1988 [41]	1988	UK	Randomized controlled trial	86	36	47	34.2	Prescribed beta-blockers 12 h before surgery	Nadolol may be recommended as a safe agent to be given before laparoscopy to reduce the frequency of arrhythmias during anesthesia.
Brady et al., 2005 [42]	2005	UK	Randomized controlled trial	103	48	55	NA	Prescribed beta-blockers before surgery and maximum 7 days	Perioperative metoprolol did not reduce 30-day cardiovascular morbidity and mortality in vascular surgical patients. No statistically or clinically significant reduction in the number of cardiovascular events in the first 30 days after surgery.
Poldermans et al., 1999 [43]	1999	UK	Randomized controlled trial	112	59	53	NA	Prescribed beta-blockers before surgery and maximum 7 days	Perioperative administration of bisoprolol reduced the incidence of both death from cardiac causes and nonfatal MI in high-risk patients undergoing major vascular surgery.
Richman et al., 2017 [44]	2017	USA	Retrospective cohort study	23,537	BB+ statin = 13,501 BB only = 2626	2346 only statin	NA	Perioperative use	Perioperative use of beta-blockers alone showed no statistical significance.
Aoyama et al., 2016 [45]	2016	Japan	Randomized controlled trial	50	25	25	NA	Intraoperative	Landiolol infusion during surgery for lung resection could not prevent AF.
Raby et al., 1998 [46]	1998	USA	Randomized controlled trial	26	11	15	NA	Prescription postoperatively for 48 h	Ischemia was predominant in the control group compared to patients treated with esmolol. Beta-blocker use was associated with low heart rate and reduced ischemia.
Ismail et al., 2020 [47]	2020	Sweden	Cohort study	2443	900	1543	NA	Prescription of beta-blockers 12 months before and after the surgery	A significant risk reduction in 90-day mortality was found in patients receiving beta-blockers.
Urban et al., 2000 [48]	2000	USA	Randomized controlled trial	109	52	55	NA	Posoperative prescription	Postoperative electrocardiographic ischemia was significantly more prevalent in the control group compared with the beta-blocker group.
Yang et al., 2006 [49]	2006	Canada	Randomized controlled trial	496	246	250	NA	Prescribed beta-blockers before surgery and during hospitalization	Intraoperative bradycardia and hypotension were more frequent in the metoprolol group. Metoprolol was not effective in reducing postoperative cardiac event rates.
Zaugg et al., 1999 [50]	1999	USA	Randomized controlled trial	63	43	20	NA	Prescribed bet a-blockers perioperatively	Patients treated perioperatively with atenolol showed improved hemodynamic stability during emergence from anesthesia and in the postoperative period. Beta-blockers used perioperatively reduced analgesic requirements and hemodynamic instability.
Zaugg et al., 2007 [51]	2007	Sweden	Randomized controlled trial	219	110	109	NA	Prescribed beta-blockers 3 h before surgery and maximum 10 days	Perioperative therapy with bisoprolol did not affect cardiovascular outcomes in elderly patients undergoing surgery with spinal block.
Bayliff et al., 1999 [52]	1999	Canada	Randomized controlled trial	99	49	50	NA	Prescribed beta-blockers before surgery and maximum 5 days	Propranolol significantly reduced the rate of treated arrhythmias in patients undergoing major thoracic surgery, from 20% to 6%.

Abbreviations: SD: standard deviation; MI: myocardial infarction; NA: not available; LVSD: left ventricular systolic dysfunction; CHF: congestive heart failure; BB: beta-blocker; AF: atrial fibrillation.

**Table 2:** Newcastle–Ottawa Scale of Cohort studies [25,26,27,28,29,30,31,36,39,40,44,47].

Author, Year	Study Design	Selection	Comparability	Outcome/Exposure	Total	Subjective Evaluation
Ahl, Rebecka, 2020 [27]	Cohort	3	2	2	7	Good Quality
McKenzie, Nicholas, 2023 [28]	Cohort	4	2	2	8	Good Quality
Ahl, Rebecka, 2018 [29]	Cohort	4	1	2	7	Good Quality
Ahl, Rebecka, 2019 [30]	Cohort	3	2	2	7	Good Quality
Ismail, Ahmad, 2021 [31]	Cohort	3	2	3	8	Good Quality
London, Martin, 2015 [25]	Cohort	4	2	3	9	Good Quality
Karapandzic, Vesna, 2007 [36]	Case Control	3	1	2	6	Good Quality
Noordjiz, Peter, 2007 [39]	Case Control	3	1	2	6	Good Quality
Park, Jungchan, 2008 [40]	Cohort	3	2	3	8	Good Quality
Richman, Joshua, 2017 [44]	Cohort	4	2	3	9	Good Quality
Ismail, Ahmad, 2020 [47]	Cohort	3	2	2	7	Good Quality
Lindenauer et al., 2005 [26]	Cohort	3	2	2	7	Good Quality

## Data Availability

The original contributions presented in the study are included in the article/Supplementary Material, further inquiries can be directed to the corresponding author/s.

## Supplementary Materials

The following supporting information can be downloaded at: https://www.mdpi.com/article/10.3390/medsci12040064/s1, Table S1: Search in PubMed; Table S2: Search in Scopus; Table S3: Search in Web of Science; Table S4: Search in Cochrane; Table S5: CINAHL Search; Table S6: CNKI Search; Table S7: Embase Search; Table S8: Table Effects Model Results by subgroup for stroke; Table S9: Leave one out analysis (Stroke); Table S10: Random Effects Model Results by subgroup for MI; Table S11: Random Effects Model Results by subgroup for MI; Table S12: Leave one out analysis for MI; Table S13: Leave one out for Bradycardia; Table S14: Random Effects Model Results by subgroup for Mortality; Table S15: Leave one out for Mortality.
