# Supplementary material for: Beta-Blocker Use in Patients Undergoing Non-Cardiac Surgery: A Systematic Review and Meta-Analysis
Source: Med Sci (Basel). 2024 Nov 11;12(4):64. doi: 10.3390/medsci12040064 (PMC11587062; doi:10.3390/medsci12040064)
Supplement: Supplementary file 1 [file medsci-12-00064-s001.zip › medsci-3207646-supplementary.pdf]

medical sciences

MDPI

# Supplementary material

**Table S1.** Search in PubMed .

| SEARCH | RESULTS |
|---|---|
| (((Intermediate risk [Mesh]) OR (Intermediate risk [Title/Abstract]) OR (RCRI [Title/Abstract]) OR (Revised Cardiac Risk Index [Mesh]) OR (Revised Cardiac Risk Index [Title/Abstract]) OR (Risk [Title/Abstract])) AND ((Non-cardiac surgery [Mesh]) OR (Non-cardiac surgery [Title/Abstract]) OR (Noncardiac surgery [Mesh]) OR (Noncardiac surgery [Title/Abstract]) OR ((non-cardiac[Title/Abstract]) AND (Surgery[Title/Abstract])) OR (surgery[Title/Abstract])) AND ((Beta blockers [Mesh]) OR (Beta blockers [Title/Abstract]) OR (Beta-adrenergic blockers [Mesh]) OR (Beta-adrenergic blockers [Title/Abstract]) OR (Beta-adrenergic antagonists [Mesh]) OR (Beta-adrenergic antagonists [Title/Abstract]))) | 1,195 |

**Table S2.** Search in Scopus .

| **SEARCH** | **RESULTS** |
|---|---|
| TITLE-ABS-KEY (Intermediate risk AND Non-cardiac surgery AND Surgery AND Beta blockers) | 360 |

**Table S3.** Search in Web of Science .

| SEARCH | RESULTS |
|---|---|
| 1: ALL=(Intermediate risk) | 62,059 |
| 2: ALL=(RCRI) | 219 |
| 3: ALL=(Revised Cardiac Risk Index) | 467 |
| 4: ALL=(Non-cardiac surgery) | 3,673 |
| 5: ALL=(Noncardiac surgery) | 8,918 |
| 6: ALL=(Beta blockers) | 50,436 |
| 7: ALL=(Beta-adrenergic blockers) | 4,994 |
| 8: ALL=(Beta-adrenergic antagonists) | 6,856 |
| 9: #1 OR #2 OR #3 | 62,557 |
| 10: #4 OR #5 | 12,048 |

| | |
|---|---|
| 11: #6 OR #7 OR #8 | 55,955 |
| 12: #9 AND #10 AND #11 | 83 |

**Table S4.** Search in Cochrane .

| SEARCH | RESULTS |
|---|---|
| #1 ((Intermediate risk OR RCRI OR "Revised Cardiac Risk Index" OR Risk)):ti,ab,kw | 305,142 |
| #2 ((Non-cardiac surgery OR Noncardiac surgery OR (non-cardiac AND surgery) OR surgery)):ti,ab,kw | 288,474 |
| #3 ((Beta blockers OR "Beta-adrenergic blockers" OR "Beta-adrenergic antagonists")):ti,ab,kw | 5,777 |
| #4 #1 AND #2 AND #3 | 234 |

**Table S5.** CINAHL Search .

| SEARCH | RESULTS |
|---|---|
| ( (MH "Intermediate Risk" OR TI Intermediate Risk OR AB Intermediate Risk OR TI RCRI OR AB RCRI OR MH "Revised Cardiac Risk Index" OR TI "Revised Cardiac Risk Index" OR AB "Revised Cardiac Risk Index" OR TI Risk OR AB Risk) ) AND ( (MH "Noncardiac Surgery" OR TI "Non-cardiac Surgery" OR AB "Non-cardiac Surgery" OR TI "Noncardiac Surgery" OR AB "Noncardiac Surgery" OR TI non-cardiac N2 surgery OR AB non-cardiac N2 surgery OR TI Surgery OR AB Surgery) ) AND ( (MH "Beta Blockers" OR TI "Beta Blockers" OR AB "Beta Blockers" OR MH "Beta-Adrenergic Blockers" OR TI "Beta-Adrenergic Blockers" OR AB "Beta-Adrenergic Blockers" OR MH "Beta-Adrenergic Antagonists" OR TI "Beta-Adrenergic Antagonists" OR AB "Beta-Adrenergic Antagonists") ) | 169 |

**Table S6.** CNKI Search .

| SEARCH | RESULTS |
|---|---|
| ((Title, Keyword and Abstract: “Intermediate risk” (Precise)) OR (Title, Keyword and Abstract: “RCRI” (Precise)) OR (Title, Keyword and Abstr act: “Revised Cardiac Risk Index” (Precise)) AND (Title, Keyword and Abstract: “Non-cardiac surgery” (Precise)) OR (Title, Keyword and Abstract: “Noncardiac surgery” (Precise)) AND ((Title, Keyword and Abstr act: “Beta blockers” (Precise)) OR (Title, Keyword and Abstract: “Beta-adrenergic blockers” (Precise)) OR (Title, Keyword and Abstract: “Beta-adrenergic antagonists” (Precise))) | 69 |

**Table S7.** EMBASE Search .

| NO. | SEARCH | RESULTS |
|---|---|---|
| #1 | 'intermediate risk' | 35,265 |
| #2 | rcri | 369 |
| #3 | 'revised cardiac risk index'/exp | 138 |
| #4 | 'revised cardiac risk index' | 623 |
| #5 | risk | 5,254,081 |
| #6 | 'non-cardiac surgery' | 3,680 |
| #7 | 'noncardiac surgery'/exp | 237 |
| #8 | 'noncardiac surgery' | 3,935 |
| #9 | 'non cardiac' | 13,857 |
| #10 | surgery | 6,413,976 |
| #11 | 'beta blockers' | 28,962 |
| #12 | 'beta-adrenergic blockers'/exp | 358,289 |
| #13 | 'beta-adrenergic blockers' | 767 |
| #14 | 'beta-adrenergic antagonists' | 391 |
| #15 | #1 OR #2 OR #3 OR #4 OR #5 | 5,254,098 |
| #16 | #9 AND #10 | 6,635 |
| #17 | #6 OR #7 OR #8 OR #16 | 10,131 |
| #18 | #11 OR #12 OR #13 OR #14 | 361,218 |
| #19 | #15 AND #17 AND #18 | 864 |

**Table S8.** Random Effects Model Results by subgroup for Stroke.

| Subgroup | k | RR | 95%-CI | $I^2$ | p-value (between) |
|---|---|---|---|---|---|
| **Year** | 10 | | | | <0.01 |
| 2020 | 1 | 1.77 | [0.44; 7.06] | -- | |

| | | | | | |
|---|---|---|---|---|---|
| 2005 | 1 | 2.62 | [0.11; 62.87] | -- | |
| 2008 | 1 | 1.93 | [1.01; 3.68] | -- | |
| 2009 | 1 | 1.33 | [0.30; 5.93] | -- | |
| 2004 | 1 | 4.97 | [0.24; 103.19] | -- | |
| 2015 | 1 | 1.35 | [1.10; 1.65] | -- | |
| 2022 | 1 | 2.79 | [1.71; 4.57] | -- | |
| 2018 | 1 | 0.86 | [0.12; 6.03] | -- | |
| 2017 | 1 | 0.86 | [0.68; 1.09] | -- | |
| 2007 | 1 | 0.99 | [0.14; 6.91] | -- | |
| **Country** | 10 | | | | 0.91 |
| Sweden | 2 | 1.45 | [0.05; 46.99] | 0.00% | |
| UK | 2 | 1.35 | [0.79; 2.31] | 0.00% | |
| Canada | 1 | 1.93 | [1.01; 3.68] | -- | |
| Netherlands | 1 | 1.33 | [0.30; 5.93] | -- | |
| Denmark | 1 | 4.97 | [0.24; 103.19] | -- | |
| USA | 2 | 1.52 | [0.00; 2743.92] | 94.40% | |
| Spain | 1 | 0.86 | [0.12; 6.03] | -- | |
| **Type of Study** | 10 | | | | 0.33 |
| Cohort Study | 5 | 1.40 | [0.74; 2.63] | 80.70% | |
| RCT | 5 | 1.81 | [1.22; 2.70] | 0.00% | |
| **Type of Surgery** | 10 | | | | <0.01 |
| Abdominal Surgery | 2 | 2.66 | [0.42; 16.62] | 0.00% | |
| Vascular Surgery | 1 | 2.62 | [0.11; 62.87] | -- | |
| Non-Cardiac Surgery | 7 | 1.17 | [0.87; 1.59] | 49.10% | |
| **Risk of Bias** | 10 | | | | 0.66 |

| | | | | | |
|---|---|---|---|---|---|
| Low Risk | 6 | 1.46 | [0.89; 2.41] | 77.90% | |
| High Risk | 3 | 1.31 | [0.54; 3.18] | 0.00% | |
| Some Concerns | 1 | 4.97 | [0.24; 103.19] | -- | |
| **Medication** | 10 | | | | 0.03 |
| Beta Blocker | 5 | 1.40 | [0.74; 2.63] | 80.70% | |
| Metoprolol | 3 | 2.03 | [1.12; 3.68] | 0.00% | |
| Bisoprolol | 2 | 1.19 | [0.19; 7.39] | 0.00% | |
| **History of CHD** | 10 | | | | <0.01 |
| Yes | 8 | 1.18 | [0.90; 1.54] | 42.30% | |
| No | 2 | 2.79 | [2.47; 3.15] | 0.00% | |
| **Chronic use of Beta Blocker** | 10 | | | | <0.01 |
| Yes | 4 | 1.13 | [0.66; 1.94] | 68.30% | |
| No | 6 | 2.20 | [1.52; 3.19] | 0.00% | |
| **History of Atrial Fibrillation** | 10 | | | | 0.53 |
| Yes | 3 | 1.74 | [0.56; 5.46] | 0.00% | |
| No | 7 | 1.43 | [0.92; 2.22] | 73.50% | |
| **History of Arrhythmia** | 10 | | | | 0.73 |
| Yes | 2 | 1.72 | [0.00; 1290.22] | 0.00% | |
| No | 8 | 1.43 | [0.97; 2.10] | 69.40% | |

**Table S9.** Leave one out analysis (Stroke) .

| **Author** | **Effect** | **Lower** | **Upper** | **I^2** | **RStudent** | **DFFITS** | **Cook's D** | **Cov. R** | **Tau^2 (del)** | **Weight** | **Is Influential** |
|---|---|---|---|---|---|---|---|---|---|---|---|
| Omitting Ahl et al., 2020 | 1.42 | 0.99 | 2.05 | 0.66 | 0.28 | -0.01 | 0.00 | 1.18 | 0.07 | 3.74 | No |

| | | | | | | | | | | | |
|---|---|---|---|---|---|---|---|---|---|---|---|
| Omitting Brady et al., 2005 | 1.43 | 1.00 | 2.04 | 0.66 | 0.37 | -0.03 | 0.00 | 1.13 | 0.07 | 0.78 | No |
| Omitting Devereaux et al., 2008 | 1.37 | 0.95 | 1.97 | 0.63 | 0.77 | 0.27 | 0.07 | 1.21 | 0.06 | 12.47 | No |
| Omitting Dunkelgrun et al., 2009 | 1.44 | 1.00 | 2.07 | 0.66 | -0.10 | -0.10 | 0.01 | 1.18 | 0.07 | 3.27 | No |
| Omitting Juul et al., 2006 | 1.41 | 1.00 | 1.99 | 0.65 | 0.80 | 0.05 | 0.00 | 1.06 | 0.06 | 0.85 | No |
| Omitting London et al., 2015 | 1.47 | 0.97 | 2.24 | 0.65 | -0.26 | -0.23 | 0.06 | 1.58 | 0.07 | 29.74 | No |
| Omitting McKenzie et al., 2023 | 1.18 | 0.93 | 1.49 | 0.35 | 2.94 | 1.67 | 1.73 | 0.51 | 0.01 | 17.06 | Yes |
| Omitting Park et al., 2018 | 1.45 | 1.02 | 2.07 | 0.66 | -0.51 | -0.14 | 0.02 | 1.13 | 0.07 | 1.99 | No |
| Omitting Richman et al., 2017 | 1.58 | 1.24 | 2.00 | 0.12 | -3.47 | -1.37 | 0.51 | 0.52 | 0.01 | 28.10 | Yes |
| Omitting Zaugg et al., 2007 | 1.45 | 1.01 | 2.07 | 0.66 | -0.37 | -0.13 | 0.02 | 1.15 | 0.07 | 2.01 | No |

**Table S10.** Random Effects Model Results by subgroup for MI .

| **Subgroup** | **k** | **RR** | **95%-CI** | **$I^2$** | **p-value (between)** |
|---|---|---|---|---|---|
| **Year** | 14 | | | | <0.01 |

| | | | | | |
|---|---|---|---|---|---|
| 1999 | 3 | 0.17 | [0.01; 2.51] | 0.0% | |
| 2005 | 1 | 0.52 | [0.13; 2.08] | -- | |
| 2008 | 1 | 0.71 | [0.58; 0.87] | -- | |
| 2009 | 1 | 0.41 | [0.20; 0.81] | -- | |
| 1997 | 1 | 3.00 | [0.13; 68.09] | -- | |
| 2004 | 1 | 0.75 | [0.17; 3.31] | -- | |
| 2007 | 2 | 0.81 | [0.00; 3121.42] | 0.0% | |
| 2015 | 1 | 1.13 | [0.95; 1.34] | -- | |
| 2022 | 1 | 3.99 | [3.00; 5.29] | -- | |
| 2006 | 2 | 0.86 | [0.10; 7.18] | 0.0% | |
| 2018 | 1 | 1.54 | [0.52; 4.53] | -- | |
| 1998 | 1 | 0.25 | [0.01; 5.54] | -- | |
| 2017 | 1 | 0.97 | [0.80; 1.18] | -- | |
| 2000 | 1 | 0.35 | [0.04; 3.28] | -- | |
| **Country** | 18 | | | | 0.56 |
| Canada | 3 | 0.72 | [0.50; 1.04] | 0.0% | |
| UK | 4 | 0.58 | [0.11; 3.11] | 55.2% | |
| Netherlands | 1 | 0.41 | [0.20; 0.81] | -- | |
| Denmark | 2 | 0.97 | [0.00; 941.50] | 0.0% | |
| Serbia | 1 | 0.51 | [0.09; 2.87] | -- | |
| USA | 5 | 0.78 | [0.12; 5.02] | 94.4% | |
| Spain | 1 | 1.54 | [0.52; 4.53] | -- | |
| Sweden | 1 | 1.98 | [0.18; 21.54] | -- | |
| **Type of Study** | 18 | | | | 0.02 |
| RCT | 13 | 0.66 | [0.51; 0.84] | 3.6% | |

| | | | | | |
|---|---|---|---|---|---|
| Cohort Study | 5 | 1.45 | [0.60; 3.54] | 94.4% | |
| **Type of Surgery** | 18 | | | | 0.58 |
| Thoracic Surgery | 1 | 0.34 | [0.07; 1.60] | -- | |
| Vascular Surgery | 4 | 0.49 | [0.09; 2.70] | 38.6% | |
| Non-Cardiac Surgery | 9 | 0.83 | [0.57; 1.20] | 64.0% | |
| Lung Surgery | 1 | 3.00 | [0.13; 68.09] | -- | |
| Abdominal Surgery | 2 | 1.71 | [0.00; 683709.19] | 81.1% | |
| Orthopaedic Surgery | 1 | 0.35 | [0.04; 3.28] | -- | |
| **Risk of Bias** | 18 | | | | 0.24 |
| Low Risk | 8 | 1.09 | [0.56; 2.11] | 93.2% | |
| High Risk | 5 | 0.56 | [0.18; 1.75] | 45.0% | |
| Some Concerns | 5 | 0.54 | [0.17; 1.73] | 0.0% | |
| **Medication** | 18 | | | | 0.18 |
| Propanolol | 1 | 0.34 | [0.07; 1.60] | -- | |
| Metoprolol | 6 | 0.72 | [0.62; 0.84] | 0.0% | |
| Bisoprolol | 3 | 0.38 | [0.01; 22.28] | 49.1% | |
| Beta Blocker | 4 | 1.61 | [0.56; 4.64] | 95.8% | |
| Atenolol | 2 | 0.28 | [0.00; 78966.23] | 35.8% | |
| Esmolol | 1 | 0.25 | [0.01; 5.54] | -- | |
| Esmolol & Metoprolol | 1 | 0.35 | [0.04; 3.28] | -- | |
| **History of CVD** | 18 | | | | 0.97 |
| Yes | 16 | 0.81 | [0.50; 1.31] | 87.7% | |
| No | 2 | 0.78 | [0.00; 4294.64] | 0.0% | |
| **History of Atrial Fibrillation** | 18 | | | | 0.57 |
| Yes | 5 | 0.66 | [0.37; 1.21] | 2.1% | |

| | | | | | |
|---|---|---|---|---|---|
| No | 13 | 0.82 | [0.44; 1.53] | 89.4% | |
| **History of Arrhythmia** | 18 | | | | 0.57 |
| Yes | 5 | 0.66 | [0.37; 1.21] | 2.1% | |
| No | 13 | 0.82 | [0.44; 1.53] | 89.4% | |
| **History of CHD** | 18 | | | | 0.47 |
| Yes | 11 | 0.74 | [0.47; 1.19] | 63.3% | |
| No | 7 | 1.02 | [0.39; 2.66] | 84.3% | |
| **Chronic use of Beta Blocker** | 18 | | | | 0.30 |
| Yes | 5 | 1.05 | [0.89; 1.23] | 0.0% | |
| No | 13 | 0.77 | [0.40; 1.46] | 90.0% | |

**Table S11.** Leave one out analysis for MI .

| Author | Effect | Lower | Upper | I^2 | RStudent | DFFITS | Cook's D | Cov. R | Tau^2 (del) | Weight | Is Influential |
|---|---|---|---|---|---|---|---|---|---|---|---|
| Omitting Bayliff et al., 1999 | 0.86 | 0.54 | 1.35 | 0.87 | -0.89 | -0.18 | 0.03 | 1.06 | 0.40 | 4.28 | No |
| Omitting Brady et al., 2005 | 0.83 | 0.52 | 1.33 | 0.87 | -0.47 | -0.06 | 0.00 | 1.12 | 0.43 | 4.92 | No |
| Omitting Devereaux et al., 2008 | 0.83 | 0.51 | 1.34 | 0.84 | -0.22 | -0.01 | 0.00 | 1.21 | 0.43 | 10.87 | No |
| Omitting Dunkelgrun et al., 2009 | 0.88 | 0.56 | 1.40 | 0.86 | -1.03 | -0.32 | 0.10 | 1.09 | 0.39 | 8.47 | No |
| Omitting Jakobsen et al., 1997 | 0.80 | 0.51 | 1.26 | 0.87 | 0.76 | 0.12 | 0.02 | 1.05 | 0.41 | 1.48 | No |

| | | | | | | | | | | |
|---|---|---|---|---|---|---|---|---|---|---|
| Omitting Juul et al., 2006 | 0.82 | 0.51 | 1.31 | 0.87 | -0.09 | 0.05 | 0.00 | 1.14 | 0.44 | 4.49 | No |
| Omitting Karapandzic et al., 2007 | 0.83 | 0.52 | 1.32 | 0.87 | -0.44 | -0.04 | 0.00 | 1.11 | 0.43 | 3.70 | No |
| Omitting London et al., 2015 | 0.78 | 0.48 | 1.27 | 0.87 | 0.52 | 0.23 | 0.06 | 1.19 | 0.42 | 10.95 | No |
| Omitting McKenzie et al., 2023 | 0.78 | 0.60 | 1.03 | 0.48 | 4.86 | 0.51 | 0.06 | 0.39 | 0.07 | 10.60 | No |
| Omitting Neary et al., 2006 | 0.83 | 0.52 | 1.32 | 0.87 | -0.38 | -0.02 | 0.00 | 1.12 | 0.43 | 4.20 | No |
| Omitting Park et al., 2018 | 0.79 | 0.49 | 1.25 | 0.87 | 0.77 | 0.23 | 0.05 | 1.10 | 0.41 | 6.28 | No |
| Omitting Poldermans et al., 1999 | 0.89 | 0.59 | 1.34 | 0.86 | -1.89 | -0.38 | 0.14 | 0.84 | 0.30 | 1.77 | No |
| Omitting Raby et al., 1998 | 0.83 | 0.53 | 1.31 | 0.87 | -0.71 | -0.06 | 0.00 | 1.06 | 0.41 | 1.50 | No |
| Omitting Richman et al., 2017 | 0.80 | 0.49 | 1.29 | 0.87 | 0.28 | 0.16 | 0.03 | 1.20 | 0.43 | 10.89 | No |
| Omitting Urban et al., 2000 | 0.84 | 0.53 | 1.32 | 0.87 | -0.65 | -0.07 | 0.01 | 1.08 | 0.42 | 2.58 | No |
| Omitting Yang et al., 2006 | 0.80 | 0.50 | 1.30 | 0.87 | 0.18 | 0.12 | 0.02 | 1.19 | 0.43 | 9.03 | No |

| | | | | | | | | | | |
|---|---|---|---|---|---|---|---|---|---|---|
| Omitting Zaugg et al., 1999 | 0.88 | 0.57 | 1.33 | 0.87 | -1.60 | -0.29 | 0.08 | 0.90 | 0.33 | 1.67 | No |
| Omitting Zaugg et al., 2007 | 0.80 | 0.51 | 1.26 | 0.87 | 0.65 | 0.14 | 0.02 | 1.07 | 0.42 | 2.32 | No |

**Table S12.** Random Effects Model Results by subgroup for Bradycardia .

| Subgroup | k | RR | 95%-CI | I² | p-value (between) |
|---|---|---|---|---|---|
| **Year** | 7 | | | | <0.01 |
| 1999 | 1 | 6.38 | [2.40; 16.98] | -- | |
| 1988 | 1 | 1.26 | [0.84; 1.88] | -- | |
| 2008 | 1 | 2.74 | [2.19; 3.43] | -- | |
| 2004 | 1 | 8.94 | [0.48; 165.61] | -- | |
| 1996 | 1 | 1.22 | [0.39; 3.88] | -- | |
| 2006 | 2 | 1.89 | [0.00; 58360.64] | 36.7% | |
| 2007 | 1 | 2.02 | [1.33; 3.09] | -- | |
| **Country** | 8 | | | | <0.01 |
| Canada | 3 | 3.08 | [1.34; 7.08] | 26.2% | |
| UK | 2 | 1.24 | [0.17; 8.80] | 0.0% | |
| Denmark | 1 | 8.94 | [0.48; 165.61] | -- | |
| USA | 1 | 1.22 | [0.39; 3.88] | -- | |
| Sweden | 1 | 2.02 | [1.33; 3.09] | -- | |
| **Type of Study** | 8 | | | | -- |
| RCT | 8 | 2.26 | [1.37; 3.74] | 64.1% | |
| **Type of Surgery** | 8 | | | | <0.01 |

| | | | | | |
|---|---|---|---|---|---|
| Thoracic Surgery | 1 | 6.38 | [2.40; 16.98] | -- | |
| Abdominal Surgery | 1 | 1.26 | [0.84; 1.88] | -- | |
| Non-Cardiac Surgery | 5 | 2.32 | [1.47; 3.67] | 23.8% | |
| Vascular Surgery | 1 | 2.83 | [1.73; 4.64] | -- | |
| **Risk of Bias** | 8 | | | | 0.30 |
| Low Risk | 2 | 3.64 | [0.02; 568.19] | 63.1% | |
| High Risk | 3 | 1.90 | [0.69; 5.22] | 69.5% | |
| Some Concerns | 3 | 1.44 | [0.08; 25.30] | 12.8% | |
| **Medication** | 8 | | | | <0.01 |
| Propanolol | 1 | 6.38 | [2.40; 16.98] | -- | |
| Nadolol | 1 | 1.26 | [0.84; 1.88] | -- | |
| Metoprolol | 3 | 2.78 | [2.16; 3.57] | 0.0% | |
| Atenolol | 2 | 1.06 | [0.01; 147.14] | 0.0% | |
| Bisoprolol | 1 | 2.02 | [1.33; 3.09] | -- | |
| **History of CVD** | 8 | | | | <0.01 |
| Yes | 6 | 2.66 | [1.68; 4.20] | 30.6% | |
| No | 2 | 1.24 | [0.17; 8.80] | 0.0% | |
| **History of Atrial Fibrillation** | 8 | | | | 0.84 |
| Yes | 3 | 2.44 | [0.61; 9.72] | 18.0% | |
| No | 5 | 2.23 | [0.93; 5.34] | 76.2% | |
| **History of Arrhythmia** | 8 | | | | 0.84 |
| Yes | 3 | 2.44 | [0.61; 9.72] | 18.0% | |
| No | 5 | 2.23 | [0.93; 5.34] | 76.2% | |
| **History of CHD** | 8 | | | | 0.92 |
| Yes | 4 | 2.41 | [1.53; 3.80] | 21.3% | |

| | | | | | |
|---|---|---|---|---|---|
| No | 4 | 2.30 | [0.54; 9.91] | 77.7% | |
| **Chronic use of Beta Blocker** | 8 | | | | 0.89 |
| Yes | 2 | 2.07 | [0.00; 140656.21] | 35.1% | |
| No | 6 | 2.33 | [1.25; 4.34] | 71.4% | |

**Table S13.** Leave one out for Bradycardia .

| **Author** | **Effect** | **Lower** | **Upper** | **I^2** | **RStudent** | **DFFITS** | **Cook's D** | **Cov. R** | **Tau^2 (del)** | **Weight** | **Is Influential** |
|---|---|---|---|---|---|---|---|---|---|---|---|
| Omitting Bayliff et al., 1999 | 2.05 | 1.35 | 3.11 | 0.61 | 1.87 | 0.53 | 0.21 | 0.64 | 0.09 | 10.23 | No |
| Omitting Burns et al., 1988 | 2.60 | 1.60 | 4.21 | 0.31 | -1.65 | -0.81 | 0.43 | 0.86 | 0.11 | 19.28 | No |
| Omitting Devereaux et al., 2008 | 2.16 | 1.13 | 4.13 | 0.60 | 0.41 | 0.19 | 0.05 | 1.56 | 0.26 | 22.00 | No |
| Omitting Juul et al., 2006 | 2.20 | 1.28 | 3.77 | 0.68 | 0.89 | 0.13 | 0.02 | 1.07 | 0.21 | 1.87 | No |
| Omitting Mangano et al., 1996 | 2.40 | 1.37 | 4.21 | 0.67 | -0.86 | -0.27 | 0.08 | 1.17 | 0.21 | 8.38 | No |
| Omitting Neary et al., 2006 | 2.32 | 1.39 | 3.87 | 0.67 | -1.10 | -0.13 | 0.02 | 0.97 | 0.18 | 1.64 | No |
| Omitting Yang et al., 2006 | 2.16 | 1.15 | 4.08 | 0.68 | 0.44 | 0.19 | 0.04 | 1.49 | 0.25 | 17.67 | No |

| | | | | | | | | | | | |
|---|---|---|---|---|---|---|---|---|---|---|---|
| Omitting Zaugg et al., 2007 | 2.33 | 1.21 | 4.46 | 0.68 | -0.22 | -0.12 | 0.02 | 1.56 | 0.27 | 18.92 | No |

**Table S14.** Random Effects Model Results by subgroup for Mortality .

| Subgroup | k | RR | 95%-CI | I² | p-value |
|---|---|---|---|---|---|
| **Year** | | | | | |
| 2020 | 1 | 0.50 | [0.42; 0.59] | -- | |
| 1999 | 2 | 0.53 | [0.00; 1122007.63] | 62.3% | |
| 2005 | 2 | 0.31 | [0.00; 25566703643.03] | 91.8% | |
| 2008 | 1 | 1.33 | [1.03; 1.73] | -- | |
| 2009 | 1 | 0.63 | [0.29; 1.36] | -- | |
| 2021 | 1 | 0.75 | [0.74; 0.77] | -- | |
| 2004 | 1 | 1.02 | [0.76; 1.38] | -- | |
| 2007 | 2 | 0.47 | [0.00; 31575.47] | 22.4% | |
| 2015 | 1 | 0.94 | [0.86; 1.04] | -- | |
| 1996 | 1 | 0.44 | [0.21; 0.91] | -- | |
| 2022 | 1 | 1.64 | [1.18; 2.27] | -- | |
| 2006 | 2 | 0.45 | [0.00; 4941.20] | 16.2% | |
| 2018 | 1 | 0.93 | [0.42; 2.08] | -- | |
| 2017 | 1 | 0.83 | [0.63; 1.10] | -- | |
| **Test for subgroup differences** | | | | | **<0.01** |
| **Country** | | | | | |
| Sweden | 3 | 0.63 | [0.33; 1.21] | 90.6% | |
| Canada | 3 | 1.00 | [0.06; 17.53] | 30.1% | |
| UK | 4 | 0.73 | [0.19; 2.74] | 43.1% | |

| | | | | | |
|---|---|---|---|---|---|
| Netherlands | 1 | 0.63 | [0.29; 1.36] | -- | |
| Denmark | 1 | 1.02 | [0.76; 1.38] | -- | |
| Serbia | 1 | 0.17 | [0.02; 1.79] | -- | |
| USA | 4 | 0.41 | [0.04; 4.70] | 99.6% | |
| Spain | 1 | 0.93 | [0.42; 2.08] | -- | |
| **Test for subgroup differences** | | | | | 0.32 |
| **Type of Study** | | | | | |
| Cohort study | 8 | 0.53 | [0.20; 1.39] | 99.9% | |
| Randomized controlled trial | 10 | 0.80 | [0.50; 1.28] | 52.4% | |
| **Test for subgroup differences** | | | | | 0.37 |
| **Type of Surgery** | | | | | |
| Abdominal surgery | 3 | 0.70 | [0.06; 8.21] | 95.1% | |
| Thoracic surgery | 1 | 2.04 | [0.19; 21.79] | -- | |
| Vascular surgery | 3 | 0.40 | [0.01; 21.80] | 53.0% | |
| Non-cardiac surgery | 10 | 0.60 | [0.29; 1.23] | 99.8% | |
| Orthopaedic surgery | 1 | 0.75 | [0.74; 0.77] | -- | |
| **Test for subgroup differences** | | | | | 0.80 |
| **Risk of Bias** | | | | | |
| Low risk | 10 | 0.63 | [0.28; 1.41] | 99.9% | |
| High risk | 5 | 0.54 | [0.16; 1.85] | 23.9% | |
| Some concerns | 3 | 0.75 | [0.24; 2.36] | 57.0% | |
| **Test for subgroup differences** | | | | | 0.80 |
| **Medication** | | | | | |
| Beta blocker | 7 | 0.57 | [0.20; 1.67] | 100.0% | |
| Propanolol | 1 | 2.04 | [0.19; 21.79] | -- | |

| | | | | | |
|---|---|---|---|---|---|
| Metoprolol | 5 | 0.90 | [0.27; 2.98] | 45.2% | |
| Bisoprolol | 3 | 0.52 | [0.10; 2.59] | 10.2% | |
| Atenolol | 2 | 0.48 | [0.05; 4.86] | 0.0% | |
| **Test for subgroup differences** | | | | | 0.56 |
| **History of CVD** | | | | | |
| Yes | 17 | 0.62 | [0.37; 1.04] | 99.9% | |
| No | 1 | 0.67 | [0.19; 2.40] | -- | |
| **Test for subgroup differences** | | | | | 0.92 |
| **History of AF** | | | | | |
| Yes | 7 | 0.32 | [0.11; 0.91] | 99.4% | |
| No | 11 | 0.99 | [0.75; 1.30] | 84.3% | |
| **Test for subgroup differences** | | | | | 0.01 |
| **History of Arrhythmic** | | | | | |
| Yes | 6 | 0.29 | [0.08; 1.05] | 98.9% | |
| No | 12 | 0.89 | [0.66; 1.21] | 87.3% | |
| **Test for subgroup differences** | | | | | 0.03 |
| **History of CHD** | | | | | |
| Yes | 13 | 0.54 | [0.30; 0.96] | 99.9% | |
| No | 5 | 1.27 | [0.49; 3.27] | 22.0% | |
| **Test for subgroup differences** | | | | | 0.04 |
| **Chronic use of Betabloker** | | | | | |
| Yes | 6 | 0.75 | [0.54; 1.04] | 89.7% | |
| No | 12 | 0.56 | [0.25; 1.25] | 99.0% | |
| **Test for subgroup differences** | | | | | 0.45 |

**Table S15.** Leave one out for Mortality .

| Author | Effect | Lower | Upper | I^2 | RStudent | DFFITS | Cook's D | Cov. R | Tau^2 (del) | Weight | Is Influential |
|---|---|---|---|---|---|---|---|---|---|---|---|
| Omitting Ahl et al., 2020 | 0.63 | 0.37 | 1.07 | 1.00 | -0.26 | -0.07 | 0.01 | 1.14 | 0.75 | 7.52 | No |
| Omitting Bayliff et al., 1999 | 0.60 | 0.37 | 1.00 | 1.00 | 0.81 | 0.13 | 0.02 | 1.05 | 0.72 | 2.47 | No |
| Omitting Brady et al., 2005 | 0.60 | 0.36 | 0.98 | 1.00 | 1.03 | 0.17 | 0.03 | 1.02 | 0.70 | 2.68 | No |
| Omitting Devereaux et al., 2008 | 0.59 | 0.35 | 0.98 | 1.00 | 0.93 | 0.26 | 0.07 | 1.09 | 0.71 | 7.42 | No |
| Omitting Dunkelgrun et al., 2009 | 0.62 | 0.37 | 1.05 | 1.00 | 0.01 | 0.00 | 0.00 | 1.13 | 0.76 | 6.20 | No |
| Omitting Ismail et al., 2021 | 0.61 | 0.36 | 1.03 | 1.00 | 0.23 | 0.07 | 0.00 | 1.15 | 0.75 | 7.60 | No |
| Omitting Juul et al., 2006 | 0.60 | 0.36 | 1.01 | 1.00 | 0.59 | 0.17 | 0.03 | 1.12 | 0.74 | 7.36 | No |
| Omitting Karapandzic et al., 2007 | 0.64 | 0.39 | 1.06 | 1.00 | -0.90 | -0.14 | 0.02 | 1.04 | 0.71 | 2.48 | No |
| Omitting Lindenauer et al., 2005 | 0.82 | 0.63 | 1.08 | 0.83 | -7.20 | -2.76 | 1.49 | 0.30 | 0.14 | 7.60 | Yes |
| Omitting London et al., 2015 | 0.60 | 0.36 | 1.01 | 1.00 | 0.50 | 0.14 | 0.02 | 1.13 | 0.74 | 7.57 | No |

| | | | | | | | | | | | |
|---|---|---|---|---|---|---|---|---|---|---|---|
| Omitting Mangano et al., 1996 | 0.64 | 0.38 | 1.07 | 1.00 | -0.39 | -0.10 | 0.01 | 1.13 | 0.75 | 6.35 | No |
| Omitting McKenzie et al., 2023 | 0.58 | 0.35 | 0.95 | 1.00 | 1.19 | 0.34 | 0.11 | 1.05 | 0.68 | 7.31 | No |
| Omitting Neary et al., 2006 | 0.62 | 0.37 | 1.04 | 1.00 | 0.07 | 0.02 | 0.00 | 1.12 | 0.76 | 4.73 | No |
| Omitting Park et al., 2018 | 0.61 | 0.36 | 1.02 | 1.00 | 0.44 | 0.11 | 0.01 | 1.12 | 0.75 | 6.15 | No |
| Omitting Poldermans et al., 1999 | 0.65 | 0.40 | 1.08 | 1.00 | -1.03 | -0.21 | 0.05 | 1.04 | 0.70 | 4.18 | No |
| Omitting Richman et al., 2017 | 0.61 | 0.36 | 1.03 | 1.00 | 0.35 | 0.10 | 0.01 | 1.14 | 0.75 | 7.39 | No |
| Omitting Yang et al., 2006 | 0.64 | 0.39 | 1.05 | 1.00 | -1.01 | -0.14 | 0.02 | 1.02 | 0.70 | 1.83 | No |
| Omitting Zaugg et al., 2007 | 0.61 | 0.37 | 1.02 | 1.00 | 0.36 | 0.07 | 0.00 | 1.09 | 0.75 | 3.17 | No |
